# Rescue Therapy after Failure of HCV Antiviral Treatment with Interferon-Free Regimens

**DOI:** 10.3390/v15030677

**Published:** 2023-03-04

**Authors:** Olga Tronina, Michał Brzdęk, Dorota Zarębska-Michaluk, Dorota Dybowska, Beata Lorenc, Ewa Janczewska, Włodzimierz Mazur, Anna Parfieniuk-Kowerda, Anna Piekarska, Rafał Krygier, Jakub Klapaczyński, Hanna Berak, Jerzy Jaroszewicz, Aleksander Garlicki, Krzysztof Tomasiewicz, Jolanta Citko, Robert Flisiak

**Affiliations:** 1 Department of Transplantation Medicine, Nephrology, and Internal Diseases, Medical University of Warsaw, 02-091 Warsaw, Poland; 2 Collegium Medicum, Jan Kochanowski University, 25-317 Kielce, Poland; 3 Department of Infectious Diseases, Voivodship Hospital, 25-317 Kielce, Poland; 4 Department of Infectious Diseases and Hepatology, Faculty of Medicine, Collegium Medicum Bydgoszcz, Nicolaus Copernicus University, 87-100 Toruń, Poland; 5 Pomeranian Center of Infectious Diseases, Medical University of Gdańsk, 80-210 Gdańsk, Poland; 6 Department of Basic Medical Sciences, School of Public Health in Bytom, Medical University of Silesia, 40-055 Katowice, Poland; 7 ID Clinic, Hepatology Outpatient Department, 41-400 Bytom, Poland; 8 Clinical Department of Infectious Diseases, Medical University of Silesia, 41-500 Chorzów, Poland; 9 Department of Infectious Diseases and Hepatology, Medical University of Białystok, 15-540 Białystok, Poland; 10 Department of Infectious Diseases and Hepatology, Medical University of Łódź, 90-419 Łódź, Poland; 11 Outpatients Hepatology Department, State University of Applied Sciences, 62-510 Konin, Poland; 12 Department of Internal Medicine and Hepatology, Central Clinical Hospital of the Ministry of Internal Affairs and Administration, 02-241 Warszawa, Poland; 13 Outpatient Clinic, Hospital for Infectious Diseases, 02-091 Warsaw, Poland; 14 Department of Infectious Diseases and Hepatology, Medical University of Silesia in Katowice, 41-902 Bytom, Poland; 15 Department of Infectious and Tropical Diseases, Jagiellonian University Collegium Medicum, 30-252 Kraków, Poland; 16 Department of Infectious Diseases, Medical University of Lublin, 20-059 Lublin, Poland; 17 Regional Hospital, 10-561 Olsztyn, Poland

**Keywords:** chronic hepatitis C, direct-acting antivirals, interferon-free, pangenotypic, rescue therapy, sustained virologic response

## Abstract

Direct-acting antivirals (DAA) regimens have provided hope for eliminating hepatitis C virus (HCV) infection. Patients following ineffective therapy with DAA, especially those previously treated with inhibitors of non-structural protein 5A (NS5A), remain a challenge. The study aimed to assess the effectiveness of DAA pangenotypic options in patients after failure of NS5A containing genotype-specific regimens. The analysis included 120 patients selected from the EpiTer-2 database with data on 15675 HCV-infected individuals treated with IFN-free therapies from 1 July 2015 to 30 June 2022 at 22 Polish hepatology centres. The majority of them were infected with genotype (GT) 1b (85.8%) and one-third was diagnosed with fibrosis F4. Among the rescue pangenotypic regimens, the most commonly used was the sofosbuvir/velpatasvir (SOF/VEL) ± ribavirin (RBV) combination. The sustained virologic response, which was a measure of treatment effectiveness, was achieved by 102 patients, resulting in cure rate of 90.3% in the per protocol analysis. All 11 non-responders were infected with GT1b, 7 were diagnosed with cirrhosis, and 9 were treated with SOF/VEL±RBV. We demonstrated the high effectiveness of the pangenotypic rescue options in patients after genotype specific NS5A-containing regimens failures, identifying cirrhosis as a negative prognostic factor of treatment effectiveness.

## 1. Introduction

Direct-acting antivirals (DAA) have revolutionised the treatment of chronic hepatitis C virus (HCV) infection, providing a chance of the virus’ eradication in 95% of patients [1]. The new therapeutic opportunities have high significance in the reduction of HCV transmission and the mortality resulting from a chronic infection, decompensated cirrhosis, and hepatocellular carcinoma (HCC) [2,3]. In addition to the high effectiveness of DAA—regardless of the genotype (GT), degree of liver fibrosis, or previous failed antiviral treatments—these new treatments are safe, short, and comfortable. The pangenotypic 8–12-week courses of pills taken once per day, resulting in low and usually tolerable side effects. Combined with the easy possibility to adjust any possible drug interactions, these treatments pave the way to effectively counteract one of the largest epidemic threats [4,5,6]. Never before have we been so close to the possibility of eliminating HCV. These new perspectives have enabled the World Health Organisation (WHO) to design a global strategy to eliminate HCV transmissions, which predicts a 90% patient identification rate leading to 80% of treated cases among the global population by 2030, which will in turn allow to reduce the mortality related to HCV complications by 65% [7].

The very high efficacy of DAAs, however, not reaching 100%, means that patients with treatment failure still occur. Chronic hepatitis C in these patients has an increased risk of disease progression, fibrosis complications, oncological risk, necessity of transplantation, or even death [8,9]. Those previously treated with regimens containing non-structural protein 5A (NS5A) inhibitors appear to be the most challenging. Virologic failure in DAAs carries the risk of the forming the resistance-associated substitutions (RASs), which can correlate with DAA resistance [10]. Prior to the era of availability of pangenotypic options, such patients were subjected to rescue retherapy with genotype-specific regimens, but the effectiveness of such management was suboptimal [11]. In such cases, RAS testing should be taken into consideration, and the retherapy should include the newest generation of DAA; although sometimes, due to limited possibilities, this may not be possible [10].

The aim of the current study was to assess the effectiveness of DAA pangenotypic options in patients after failure of genotype-specific regimens containing NS5A inhibitors.

## 2. Materials and Methods

### 2.1. Study Population

The EpiTer-2 observational study, initiated by the Polish Association of Epidemiologists and Infectiologists gathered 22 centres, which were conducting treatments of chronic HCV infection in real world settings (https://www.epiter-2.pl/ (accessed on 20 November 2022). The data collected from 1 July 2015 to 30 June 2022, using a web-based questionnaire, allowed to assess the effectiveness of pangenotypic treatment and the safety of patients who failed previous DAA-based genotype-specific therapy (Table 1).

The treatment was conducted and financed in accordance with the therapeutic program of the National Health Fund. Apart from the basic patient data, such as age, sex, BMI, comorbidities, history of hepatocellular carcinoma, history of liver decompensation: encephalopathy, oesophageal varices and ascites, the database also gathered data on the HCV infection—genotype, viral load, degree of hepatic fibrosis and history of antiviral treatment.

The degree of liver fibrosis was measured with non-invasive methods: transient elastography using FibroScan (Echosens, Paris, France), or shear wave elastography using Aixplorer (Supersonic, Aix- en- Provence, France). According to the European Association for the Study of the Liver recommendations, cut-offs for F3 and F4 Fibroscan test were 10 kPa and 13 kPa and for Aixplorer test 9 and 13 kPa, respectively [4]. Before the start of treatment, the HCV GT and viral load were assessed using, depending on the availability, one of the two analysers: Roche COBAS TaqMan or Abbott RealTime, both with an LLOQ of 15 IU/mL. HCV ribonucleic acid (RNA) was tested at the end of treatment and 12 weeks after its completion to assess sustained virologic response (SVR), a measure of the effectiveness of therapy. The selection of the antiviral regimen was made by the treating physicians based on current national recommendations and the reimbursement policy by the National Health Fund (NHF) [12]. Data on the course of therapy, deaths, adverse events, including severe ones, were collected during the treatment duration and 12-week follow-up to assess the safety of the therapy.

### 2.2. Ethical Considerations

In accordance with pharmaceutical regulations (Pharmaceutical Law of 6 September 2001, art. 37al), treatments conducted as a part of the National Health Fund’s therapeutic program, using registered drugs, and not endangering the patient with additional interventions beyond the scope of the program, do not require the consent of the Bioethical Committee. All data regarding the patients were catalogued in an encrypted database accessible only by the attending physician.

### 2.3. Statistical Analysis

All statistical analyses were carried out with Statistica v. 13 (StatSoft, Tulsa, OK, USA). Categorical data were expressed by frequencies and percentages, whereas continuous data were summarized by median, IQR (interquartile range) as well as minimum and maximum values. Group comparisons were performed with Pearson’s χ^2^ test or Fisher exact test (as appropriate) for categorical data and the Mann–Whitney test for continuous. *p* values of less than 0.05 were considered to be statistically significant. Per-protocol (PP) analysis concerned patients who had HCV RNA evaluation after 12 weeks from the end of treatment.

## 3. Results

### 3.1. Characteristics of the Study Group

From a total of 15,675 patients treated for chronic HCV, all 120 patients with a history of failed genotype-specific DAA therapy who received pangenotypic retherapy were selected from the EpiTer database. Reinfection was excluded based on history analysis and risk assessment. The data on these 120 patients can be found in Table 2. The majority of the patients were male (72.5%), and the median age was 53. The median BMI was 26.7, more than half of the patients had comorbidities, and 65.8% were taking concomitant medications. Reference values for laboratory parameters are presented in Table S1 Supplementary.

In the entire EpiTer-2 population (*n* = 15,675) the majority of patients were infected with GT1b (75.5%) followed by GT3 (12.9%), GT4 (4.9%), GT1a (4.3%), and other genotypes (2.4%).

The majority of the analysed 120 patients (93.3%) were infected with HCV GT1 (1b 85.8%, 1a 7.5%), while none of the patients were infected with GT 2, 5, and 6 HCV (Table 2). In total, the patients with liver cirrhosis (F4 = 35%) and advanced liver fibrosis (F3 = 14.2%) constituted nearly 50% of the treatment group (Table 3).

Nine patients (7.5%) had a history of liver decompensation in the past, with ascites (5.8%) and/or encephalopathy (1.7%). None of the patients had hepatocellular carcinoma in their history, and only one underwent a liver transplant in the past. An HBV coinfection with a positive HBs antigen and HIV was identified in 1.7% and 10.8% of patients, respectively (Table 2). Despite the fact that one in three patients was diagnosed with cirrhosis, in 83.3% of cases, at the time of the antiviral treatment’s commencement, cirrhosis was compensated.

### 3.2. Treatment Regimens

All patients were treated in the past with a DAA regimen containing a NS5A inhibitor, 32.5% with Ombitasvir (OBV), 30% with Ledipasvir (LDV), 27.5% with Elbasvir (EBR), and 10% with Daclatasvir (DCV), in most cases (64.2%) reaching end treatment response (ETR) but not SVR (Table 4). Two GT3-infected patients had previously failed to respond to LDV/SOF therapy. During retherapy, 61.7% of patients were administered Sofosbuvir/Velpatasvir (SOF/VEL) with or without RBV, while 28.3% of patients were administered Glecaprevir/Pibrentasvir (GLE/PIB).

### 3.3. Antiviral Treatment Effectiveness

**Figure 1:**
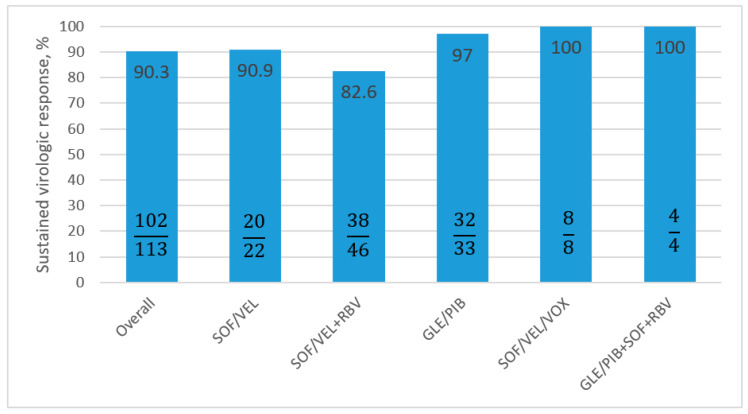
Treatment effectiveness according to regimen, calculated as PP analysis. PP: per protocol; SOF: Sofosbuvir; VEL: Velpatasvir; RBV: Ribavirin; GLE: Glecaprevir; PIB: Pibrentasvir; VOX: Voxilaprevir.

In total, 102 of the 113 patients (90.3%) achieved SVR 12 in the per protocol analysis (Figure 1).

The effectiveness of the most commonly selected treatment (SOF/VEL±RBV) was 11.7% lower than the (GLE/PIB) regimen—SVR 12 PP 97%. The addition of RBV to the SOF/VEL regimen had no influence on the increase in virologic response; SVR PP in the SOF/VEL therapy was 90.9% and for SOF/VEL+RBV was 82.6%

In two antiviral options, GLE/PIB+SOF+RBV and SOF/VEL/Voxilaprevir (VOX), 100% effectiveness was obtained in PP analyses; although, it needs to be underlined that the patient counts in both groups were low, four and eight patients, respectively.

In total, 36 patients achieved SVR in the GLE/PIB regimen (including 4 with additional SOF + RBV). Only one patient with GT1b and cirrhosis completed the GLE/PIB treatment with negative viremia, but she did not obtain SVR.

Out of the patients assessed 12 weeks after the end of treatment (113/120), only eleven did not reach SVR—six males and five females (Table 5). The remaining seven patients were lost to follow-up. All of the non-SVR patients were infected with the GT1b, seven (64%) had liver cirrhosis, ten were treated with the SOF/VEL regimen, including eight with additional RBV. All of the above-mentioned patients qualified for the 12-week therapy. One patient discontinued the treatment during the second week due to his own decision. A second patient discontinued the treatment during the fourth week due to adverse effects related to the digestive tract. The remaining patients declared they were taking the medications in accordance with the recommendations.

In the context of the entire group, the virological response in patients who were administered RBV was lower by 10%. It needs to be stressed, however, that in the group treated with RBV, 41.5% suffered from liver cirrhosis, while in the group treated without RBV, that proportion was only 29.8%.

**Figure 2:**
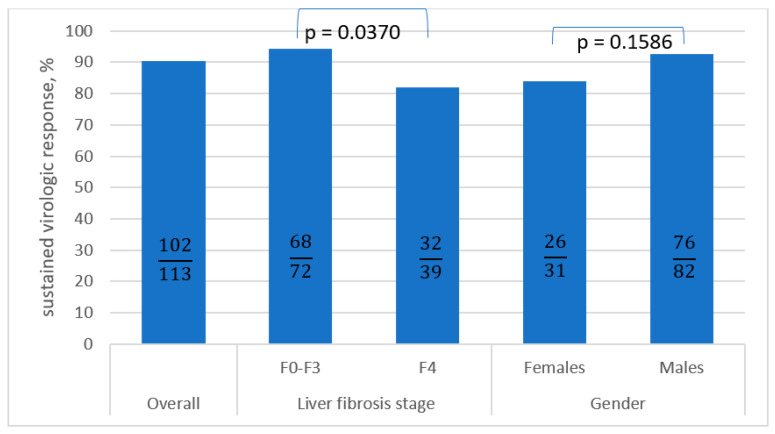
The comparison of SVR in the subpopulations calculated as PP analysis ^a^. SVR: sustained virologic response; PP: per protocol; F: fibrosis. ^a^ Pearson’s chi-squared test was used.

Comparing patients who responded to antiviral treatment to the virologic non-responders, the following differences were noted: older age and liver cirrhosis were noted as potential factors for the lack of response to treatment (Table 6, Figure 2). However, only liver cirrhosis and use of SOF/VEL±RBV in current treatment reached statistical significance (*p* ≤ 0.05).

### 3.4. Safety

Twenty-eight (23%) patients reported at least one adverse effect. The most frequent, occurring in >2% of patients, was fatigue and weakness reported by 12 patients (10%), and anaemia (2.5%). The majority of patients completed the treatment course according to the schedule, while three persons (2.5%) discontinued the antiviral treatment due to adverse effects. Of these, two did not achieve SVR. Serious adverse events occurred in four patients (3.3%). In one patient, ascites appeared; one had encephalopathy, while another one had hepatocellular carcinoma diagnosed during the follow-up period, which was not identified before and during treatment. One patient died during therapy due to liver decompensation.

None of the patients had liver decompensation that would urgently qualify them for a transplant. In the only patient who had liver transplantation, their function was assessed as stable during and after treatment (Table 7).

## 4. Discussion

Direct-acting antiviral treatment in HCV-infected patients who have not been treated before, treated with interferon with RBV or triple therapy with first-generation DAA allows to obtain nearly cure rate, reducing the risk of transmission and complications resulting from the progression of liver fibrosis, and contributing the HCV-related mortality [13,14,15,16]. A significant issue appears in patients previously treated with new generation DAA, who did not respond to treatment and have complex factors worsening the response to retherapy, such as liver cirrhosis, infection with GT3, hepatocellular carcinoma, or multiple NS5A resistant variants [17,18,19,20].

Although the hepatologic associations define therapeutic recommendations for the most complicated populations of HCV infected patients, it seems that there is still no clear optimal strategy for retherapy. This is often the result of the lack of access to specific therapeutic options, drug combinations, or long waiting times for the treatment, all resulting from the limitations of reimbursement policy. These limitations in the availability of recommended retreatment options mean that patients with advanced liver fibrosis with risk factors for disease progression, who are unsuccessfully treated with genotype-specific combinations, are being retreated with the same type of regimen. The effectiveness of genotype-specific options after failure of IFN-free therapy was evaluated in a real-world experience (RWE) cohort of 31 Polish patients, 71% of whom were cirrhotic and 74% who were infected with GT1b HCV [11]. Among the regimens used in the primary course of antiviral treatment, the combination of OBV/PTV/r±DSV±RBV was most often used, and the LDV/SOF±RBV option was most common in retherapy. The effectiveness of genotype-specific rescue therapy was shown to be 86%, which was considered moderate. Opportunities for higher effectiveness in the DAA-nonresponder population have emerged with the availability of pangenotypic regimens, as was the case in Poland in 2018. According to the guidelines of the American Association for the Study of Liver Diseases (AASLD) and the European Association for the Study of the Liver (EASL), patients with a history of failed DAA failure, including treated with NS5A inhibitors, should be retreated with SOF/VEL/VOX for 12 weeks or GLE/PIB for 16 weeks (AASLD). European guidelines additionally allow the use of GLE/PIB in combination with SOF for 12 weeks, or therapies extended to 16 or 24 weeks with the addition of RBV in liver cirrhosis [4,6]. The recommendations regarding SOF/VEL/VOX stem from the POLARIS-1 study, in which patients who failed NS5A inhibitor therapies, treated for 12 weeks with SOF/VEL/VOX, obtained a 96% virological response; overall, 34% of these patients had liver cirrhosis [21]. Although in our observation only eight (6.7%) patients (as a result of delayed availability of the drug in the therapeutic program) were treated with this regimen, they all obtained SVR. In the aforementioned POLARIS-1 study, 100% of patients infected with the GT1b, which is the most common in our population, responded to antiviral treatment [21].

The data analysis from the RWE Veterans Affairs HCV Clinical Case Registry and presented by Belperio et al. confirms a significantly high virological response of SOF/VEL/VOX. Patients with a history of failed genotype-specific options treatments, who completed the SOF/VEL/VOX therapy, achieved a 94 to even 100% SVR [22]. Equally impressive results, with 96% SVR in RWE observation, were presented by Degasperi et al. from NAVIGATORE-Lombardia and Veneto Study Groups [20]. The study was from 27 Italian centres and was based on the treatment of 179 patients with previous failed DAA and 44% cirrhosis rate. The virological response for individual genotypes did not vary significantly, and patients with genotype 1 obtained 97% SVR 12.

In our study, all patients responded to SOF/VEL/VOX therapy, although data from a meta-analysis by Xie et al. from a Canadian centre indicated that this option was less effective in patients previously treated with SOF/VEL [23]. The 12-week SOF/VEL/VOX therapy may be an effective option not only for patients with failed genotype-specific therapy, but also for those who failed to achieve SVR 12 on the pangenotypic GLE/PIB regimen. Thirty-one such cases were documented by Pearlman et al. The overall SVR reached 94%, with 17 of 18 patients diagnosed with cirrhosis [24]. However, the SOF/VEL/VOX regimen recommended as a rescue option for patients after ineffective therapy with an NS5A inhibitor became available in Poland only in May 2021. Therefore, patients in the analysed cohort were treated with other pangenotypic regimens, the most commonly used of which was the SOF/VEL combination, with or without RBV. This regimen was administered to 74 patients, accounting for 61.7% of the analysed population.

The effectiveness and safety of SOF/VEL therapy in patients not previously treated with an NS5A inhibitor is not in doubt, regardless of GT or the presence of cirrhosis. This is supported by a multicentre analysis conducted in several countries in Europe and North America, in which more than 5000 patients treated for 12 weeks without RBV achieved an overall SVR of 92.6%, with only 1% virological failures [25]. Our analysis showed that for patients after failure of NS5A inhibitor-containing therapy, this option is less effective with a documented SVR of 85.3%. It should be noted that 80% of those treated with SOF/VEL had liver cirrhosis or advanced liver fibrosis. An alternative in the SOF/VEL combination, increasing the virological response, could be the individualization and extension of the therapy to 24 weeks. According to Gane’s et al. observations, 49 of the 51 (96%) patients without cirrhosis and with a history of failed SOL/VEL therapy who were administered SOF/VEL for 24 weeks obtained SVR, including 100% (*n* = 5) with GT1b [26]. Patients infected with GT3 presented a significantly worse response than the remaining genotypes, with only 78% of virological response. A combined occurrence of adverse factors in patients with genotype 3, cirrhosis, and VEL-specific resistance-associated substitutions (RASs) clearly causes the deterioration of the virologic response. In the second and third phase of clinical trials, the patients reached an SVR of 80%, or even 57% in the case of Y93H. The addition of ribavirin increased the SVR to 95% and 89%, respectively.

Further studies with a much larger group of treated patients would be useful. However, the possibility of SOF/VEL/VOX and GLE/PIB treatment makes it unlikely that studies analysing the efficacy of SOF/VEL therapy with the addition of RBV will be conducted.”

The second most often chosen therapeutic regimen in our research was GLE/PIB, applied to 34 patients (28.3%). The treatment time, in accordance with the National Health Fund program, was 12 weeks, which diverts from the AASLD guidelines recommending 16-week therapy. However, the available data from the meta-analysis by Shen et al., summarizing the effectiveness and safety of GLE/PIB in patients with a history of ineffective DAA therapy, documents the lack of statistically significant difference in virological response for 12- and 16-week treatment with an overall SVR of 96.8% [27]. Most of the reports included in this meta-analysis were carried out in the Japanese population, with only two studies (two out of the three with a 16-week duration) with a total of 268 patients being from the U.S., with an SVR lower by 6.8% (91.1 vs. 97.9%). This was used to explain the lack of SVR difference in 12- and 16-week options in relation to the entire population, while the sub-population analysis showed a higher percentage of SVR in the 16-week regimen. These data are confirmed by the MAGELLAN-1 Part 2 study, in which 47 patients treated with GLE/PIB for 16 weeks reached SVR in 94% of cases compared to 89% in patients with a 12-week therapy [28]. In our study, only one patient treated with GLE/PIB for 12 weeks did not achieve SVR; it was a woman with cirrhosis infected with GT1b.

The most frequent factor associated with the failure of antiviral therapy is liver cirrhosis, especially in the case of decompensation [29]. Although this is denied by the meta-analysis by Shen et al. regarding GLE/PIB therapy (SVR 95.3% with cirrhosis and 96.3% with non-cirrhosis), many researchers are in agreement regarding this issue [27,30]. This is also confirmed by the current analysis. Our patients with fibrosis F0-F3 reached SVR in 94.4% of cases (PP), in comparison to 82.1% in those with cirrhosis. In the Spanish cohort presented by Llaneras et al., despite an excellent 95% SVR to SOF/VEL/VOX treatment, including 100% in case of GT1b-infected patients, those with cirrhosis (34% out of 137 infected patients) eradicated the virus in 89% of cases in comparison to patients without cirrhosis (98%) [31].

In addition to being highly effective, DAA therapies are also safe, as our study has also documented. Adverse effects, even if reported seemingly frequently, in most cases are not significant clinically [24]. The profile and percentage of adverse effects occurring in the group treated by us varies slightly from those reported by other researchers. The total number of patients reporting adverse effects is lower (23.3% vs. >40%), which could be the result of not reporting the less inconvenient effects [27,28,32]. The most common adverse event, reported by 10% of patients, was fatigue. Headaches, nausea, diarrhoea, and itchiness were reported in less than 2% of cases, while in other studies these numbers reached 4–10% [23]. Anaemia was reported in three of our patients, and in all cases was related to the selection of the therapeutic option with RBV. The role and safety of RBV in the treatment of chronic hepatitis C remains a matter of debate. In a study conducted by Lok et al., the use of ribavirin led to dose reduction or treatment discontinuation in 38% of patients, with little or no effect on treatment efficacy [32]. On the other hand, data from the Chronic Hepatitis Cohort Study, which includes more than 4000 patients treated with various DAA options of all generations with or without RBV, published by Lu et al., clearly shows that in patients with a history of failed DAA (contrary to those with a history of interferon therapy) with decompensated liver cirrhosis (contrary to patients without cirrhosis or with compensated cirrhosis) and infected with GT3 (contrary to GT1 and 2), the addition of RBV to the therapy increases the SVR percentage [33]. Our analysis documents not only the lack of improvement in the effectiveness of therapy by adding RBV, but even its worsening, which may be influenced by the higher severity of liver disease in patients treated with an RBV-containing regimen. It should be noted, however, that according to the AASLD recommendations, the addition of RBV in retherapy is an option recommended in particularly difficult cases of previous therapeutic failures, multiple DAA treatments, and in patients infected with HCV GT3 and/or with cirrhosis. As alternative methods of improving the SVR in this population of patients, it is recommended to extend the duration of treatment or use the triple GLE/PIB+SOF regimen [6].

### Limitations

The main limitation of this work is the uniformity of the groups treated with antiviral treatment, which does not allow us to form conclusions regarding antiviral response for patients with GT other than 1b. In the studied group, only 7.5% of patients had GT1a, 4.2% had GT4, and 1.7% had GT3.

Another limitation is also the low number of patients, especially in the options recommended by hepatologic associations for patients with a history of NS5A DAA failures. Limitations typical of retrospective RWE studies should be also mentioned, including possible bias, data entry errors, and underreporting of the adverse events, especially those mild in intensity. In selected cases, especially with a repeated history of NS5A inhibitors therapy failures, resistance-associated substitutions (RASs) testing could optimize the choice of therapy. Such tests were not performed in our patients, which is also a limitation of the study. The knowledge of the resistance profile could help to estimate the probability of a virological answer, and could also differentiate between virological relapse and reinfection. RASs testing is not obligatory, but is recommended by hepatological societies [4].

Despite a few limitations, the work has one significant strength. The patients included in the study received treatment in real-world conditions, with the options that were currently available. In light of no other therapeutic possibilities being available, the most seriously ill patients—those with advanced liver fibrosis and cirrhosis—received the possibility of eradicating the virus and stopping the progression of the disease.

## 5. Conclusions

Our study documented the high effectiveness of pangenotypic regimens in patients who had previously failed a genotype-specific DAA regimen containing an NS5A inhibitor. The presence of liver cirrhosis was identified as a negative prognostic factor of treatment effectiveness in the univariate analysis.

## Figures and Tables

**Table 1:** Genotype specific and pangenotypic regimens and their composition.

Regimen	NS3 Inhibitor	NS5A Inhibitor	NS5B Inhibitor
Genotype-specific			
ASV+DCV	asunaprevir	daclatasvir	
LDV/SOF±RBV		ledipasvir	sofosbuvir
OBV/PTV/r±DSV±RBV	paritaprevir	ombitasvir	dasabuvir
GZR/EBR±RBV	grazoprevir	elbasvir	
Pangenotypic			
GLE/PIB	glecaprevir	pibrentasvir	
GLE/PIB+SOF+RBV	glecaprevir	pibrentasvir	sofosbuvir
SOF/VEL±RBV		velpatasvir	sofosbuvir
SOF/VEL/VOX	voxilaprevir	velpataswir	sofosbuvir

NS, nonstructural.

**Table 2:** Baseline characteristics.

Parameter	All Patients, *n* = 120
Gender, females/males, *n*(%)	33 (27.5)/87 (72.5)
Age [years], median (IQR)	53 (40–63.5)
Females	62 (58–69)
Males	49 (38–61)
BMI, median (IQR)	26.7 (23.8–29.7)
Comorbidities, *n*(%)	
Any comorbidity	72 (60)
Hypertension	39 (32.5)
Diabetes	11 (9.2)
Renal disease	6 (5)
Autoimmune diseases	2 (1.7)
Non-HCC tumours	3 (2.5)
Other	47 (39.2)
Concomitant medications, *n*(%)	79 (65.8)
ALT IU/L, median (IQR)	59 (40.4–97.5)
Bilirubin mg/dL, median (IQR)	0.6 (0.5–1)
Albumin g/dL, median (IQR)	4.1 (3.8–4.5)
Creatinine mg/dL/GFR (ml/min)/1.73 m^2^, median (IQR)	0.9 (0.7–1)/98.6 (88.1–111)
Haemoglobin g/dL, median (IQR)	14.8 (14–16)
Platelets, ×1000/µL, median (IQR)	173 (120–233.5)
HCV RNA ×10^6^ IU/mL, median (IQR)	1.5 (0.6–3.7)

SD: Standard deviation; BMI: Body mass index; HCC: Hepatocellular carcinoma; ALT: Alanine transaminase; HCV: Hepatitis C virus.

**Table 3:** Characteristics of liver disease.

Parameter	All Patients, *n* = 120
GT, *n*(%)	
1	0
1a	9 (7.5)
1b	103 (85.8)
2	0
3	2 (1.7)
4	5 (4.2)
5	0
6	0
Mixed genotype	1 (0.8)
Liver fibrosis, *n*(%)	
F0	1 (0.8)
F1	32 (26.6)
F2	26 (21.7)
F3	17 (14.2)
F4	42 (35)
No data	2 (1.7)
Child-Pugh, *n*(%)	
B	6 (5)
C	1 (0.8)
History of hepatic decompensation, *n*(%)	
Ascites	7 (5.8)
Encephalopathy	2 (1.7)
Documented oesophageal varices, *n*(%)	11 (9.2)
Hepatic decompensation at baseline, *n*(%)	
Moderate ascites—responded to diuretics	5 (4.2)
Tense ascites—not responded to diuretics	0
Encephalopathy	1 (0.8)
HCC history, *n*(%)	0
OLTx history, *n*(%)	1 (0.8)
HBV coinfection (HBsAg+), *n*(%)	2 (1.7)
HIV coinfection, *n*(%)	13 (10.8)

GT: Genotype; F: Fibrosis; MELD: Model End-Stage Liver Disease; HCC: Hepatocellular carcinoma; OLTx: Orthotopic liver transplantation; HBV: Hepatitis B virus; HBsAg+: Hepatitis B surface antigen; HIV: Human immunodeficiency virus.

**Table 4:** Treatment characteristics.

Parameter	All Patients, *n* = 120
History of previous therapy, *n*(%)	
Nonresponder	31 (25.8)
Relapser	77 (64.2)
Discontinuation due to safety reason	5 (4.2)
Unknown type of response	7 (5.8)
Number of patients with previous treatment failure, *n*(%)	
ASV+DCV	12 (10)
LDV/SOF±RBV	36 (30)
OBV/PTV/r±DSV±RBV	39 (32.5)
GZR/EBR±RBV	33 (27.5)
Current treatment regimen, *n*(%)1 July 2015–30 June 2022	
GLE/PIB	34 (28.3)
GLE/PIB+SOF+RBV	4 (3.3)
SOF/VEL±RBV	74 (61.7)
SOF/VEL+RBV	49 (40.9)
SOF/VELMay 2021–30 June 2022	25 (20.8)
SOF/VEL/VOX	8 (6.7)

ASV: Asunaprevir; DCV: Daclatasvir; LDV: Ledipasvir; SOF: Sofosbuvir; RBV: Ribavirin; OBV: Ombitasvir; PTV/r: Paritaprevir; DSV: Dasabuvir; GZR: Grazoprevir; EBR: Elbasvir; GLE: Glecaprevir; PIB: Pibrentasvir; VEL: Velpatasvir; VOX: Voxilaprevir.

**Table 5:** Characteristics of 11 Virologic Failures to Treatment.

Patient	Age	GT	F, CP	Current Regimen	History of Previous Therapy	Baseline HCV RNA × 10^6^ IU/mL	Treatment Course	EOT
Male 1	64	1b	4, A	SOF/VEL+RBV	Relapser, LDV/SOF±RBV	0.276	according to schedule	TD
Male 2	43	1b	2, A	SOF/VEL+RBV	Relapser, LDV/SOF±RBV	0.878	therapy discontinuation	TND
Male 3	62	1b	2, A	SOF/VEL	Relapser, GZR/EBR	3.88	according to schedule	TD
Male 4	58	1b	4, A	SOF/VEL+RBV	Relapser, LDV/SOF±RBV	6.7	according to schedule	TD
Male 5	40	1b	4, A	SOF/VEL+RBV	Relapser, LDV/SOF±RBV	0.907	according to schedule	TD
Male 6	61	1b	4, A	SOF/VEL	Relapser, OBV/PTV/r+DSV±RBV	0.495	according to schedule	TND
Woman 1	67	1b	3, A	SOF/VEL+RBV	Discontinuation due to safety reason, LDV/SOF±RBV	2.63	therapy discontinuation	TND
Woman 2	61	1b	4, A	SOF/VEL+RBV	Relapser, LDV/SOF±RBV	0.513	according to schedule	TD
Woman 3	63	1b	4, A	SOF/VEL+RBV	Relapser, ASV+DCV	1.3	according to schedule	TD
Woman 4	71	1b	4, A	GLE/PIB	Nonrespoder, GZR/EBR	7.32	according to schedule	TND
Woman 5	69	1b	3, A	SOF/VEL+RBV	Nonrespoder, GZR/EBR	6.04	according to schedule	TND

GT: genotype; F fibrosis; CP: Child-Pugh; HCV: Hepatitis C virus; EOT: end of therapy; SOF: Sofosbuvir; VEL: Velpatasvir; RBV: Ribavirin; GLE: Glecaprevir; PIB: Pibrentasvir; LDV: Ledipasvir; GZR: Grazoprevir; EBR: Elbasvir; OBV: Ombitasvir; PTV/r: Paritaprevir; DSV: Dasabuvir; TD: targed detected = HCV RNA detectable; TND: target not detected = HCV RNA undetectable.

**Table 6:** The Comparison of Virological Responders and Non-Responders to Antiviral Therapy.

Parameter	Responders, *n* = 102	Non-Responders, *n* = 11	*P* ^1^
Gender, females/males, *n*(%)	26 (25.5)/76 (74.5)	5 (45.5)/6 (54.5)	0.1585 ^2^
Age [years], median (IQR)	51.5 (40–63)	62 (58–67)	0.0533 ^3^
Females	61 (43–70)	67 (63–69)	0.2400 ^3^
Males	48.5 (37.5–58)	59.5 (43–62)	0.3920 ^3^
BMI, median (IQR)	26.7 (23.9–29.4)	29.3 (25.4–33.5)	0.2279 ^3^
Current treatment regimen, *n*(%)			
GLE/PIB	32 (31.4)	1 (9.1)	0.1115
GLE/PIB+SOF+RBV	4 (3.9)	0	0.6600
SOF/VEL±RBV	58 (56.9)	10 (90.9)	0.0251
SOF/VEL	20 (19.6)	2 (18.2)	0.6360
SOF/VEL+RBV	38 (37.3)	8 (72.7)	0.0262
SOF/VEL/VOX	8 (7.8)	0	0.4286
GT, *n*(%)			
GT1b	87 (85.3)	11 (100)	0.1720 ^2^
non-GT1b	15 (14.7)	0	
Comorbidities, *n*(%)			
Any comorbidity	61 (59.8)	7 (63.6)	0.8051 ^2^
Hypertension	36 (35.3)	3 (27.3)	0.4332
Diabetes	9 (8.8)	1 (9.1)	0.6569
Renal disease	6 (5.9)	0	0.5330
Autoimmune diseases	2 (2)	0	0.8140
Non-HCC tumours	1 (1)	1 (9.1)	0.1860
Other	41 (40.2)	3 (27.3)	0.3116
Concomitant medications, *n*(%)	64 (62.7)	9 (81.8)	0.2088 ^2^
History of previous therapy, *n*(%)			
Nonresponder	26 (25.5)	2 (18.2)	0.4551
Relapser	69 (67.6)	8 (72.7)	0.5127
Discontinuation due to safety reason	2 (2)	1 (9.1)	0.2667
Unknown type of response	5 (4.9)	0	0.5934
Number of patients with treatment failure, *n*(%)			
ASV+DCV	11 (10.8)	1 (9.1)	0.6699
LDV/SOF±RBV	26 (25.5)	6 (54.5)	0.0513
OBV/PTV/r±DSV±RBV	35 (34.3)	1 (9.1)	0.0794
GZR/EBR±RBV	30 (29.4)	3 (27.3)	0.6212
History of hepatic decompensation, *n*(%)			
Ascites	4 (3.9)	2 (18.2)	0.1044
Encephalopathy	1 (1)	0	0.9026
Documented oesophageal varices, *n*(%)	10 (9.8)	1 (9.1)	0.7095
Hepatic decompensation at baseline, *n*(%)			
Moderate ascites—responded to diuretics	3 (2.9)	0	0.7333
Tense ascites—not responded to diuretics	0	0	NA
Encephalopathy	0	0	NA
HCC history, *n*(%)	0	0	NA
OLTx history, *n*(%)	1 (1)	0	0.9026
Liver fibrosis, *n*(%)			
F0-F3	68 (68.6)	4 (36.4)	0.0424
F4	32 (31.4)	7 (63.6)	
No data	2 (2)	0	
Child-Pugh, *n*(%)			
B	5 (4.9)	0	0.4526 ^2^
C	0	0	NA
HBV coinfection (HBsAg+), *n*(%)	2 (2)	0	0.8140
HIV coinfection, *n*(%)	10 (9.8)	1 (9.1)	0.7095
ALT IU/L, median (IQR)	59 (40.7–99)	62 (35–76)	0.6913 ^3^
Bilirubin mg/dL, median (IQR)	0.6 (0.5–1)	0.8 (0.5–1)	0.6877 ^3^
Albumin g/dL, median (IQR)	4.1 (3.9–4.5)	3.9 (3.6–4.2)	0.1969 ^3^
Creatinine mg/dL, median (IQR)	0.9 (0.7–1)	0.8 (0.7–1)	0.3232 ^3^
Haemoglobin g/dL, median (IQR)	15.2 (14–16)	14 (13.2–15.9)	0.1782 ^3^
Platelets, ×1000/µL, median (IQR)	173 (127–234)	188 (84–237)	0.6247 ^3^
HCV RNA ×10^6^ IU/ml, median (IQR)	1.6 (0.6–3.6)	1.3 (0.5–6)	0.7751 ^3^

SD: Standard deviation; BMI: Body mass index; GLE: Glecaprevir; PIB: Pibrentasvir; SOF: Sofosbuvir; RBV: Ribavirin; VEL: Velpatasvir; VOX: Voxilaprevir; GT: genotype; HCC: Hepatocellular carcinoma; ASV: Asunaprevir; DCV: Daclatasvir; LDV: Ledipasvir; Ombitasvir; PTV/r: Paritaprevir; DSV: Dasabuvir; GZR: Grazoprevir; EBR: Elbasvir; OLTx: Orthotopic liver transplantation; F: Fibrosis; MELD: Model End-Stage Liver Disease; HBV: Hepatitis B virus; HBsAg: Hepatitis B surface antigen; HIV: Human immunodeficiency virus; ALT: Alanine transaminase; HCV: Hepatitis C virus; IQR: interquartile range. ^1^ Fisher’s exact test was used unless otherwise noted. ^2^ Pearson’s chi-squared test was used. ^3^ Mann–Whitney U test was used.

**Table 7:** Safety of antiviral therapy.

Parameter	All Patients, *n* = 120
Treatment course, *n*(%)	
according to schedule	109 (90.8)
therapy modification (including RBV dose)	5 (4.2)
therapy discontinuation	3 (2.5)
No data	3 (2.5)
Patients with at least one AE, *n*(%)	28 (23.3)
Serious adverse events, *n*(%)	4 (3.3)
AEs leading to treatment discontinuation, *n*(%)	3 (2.5)
Most common AEs (≥2%), *n*(%)	
weakness/fatigue	12 (10)
Anaemia	3 (2.5)
AEs of particular interest (cirrhotic), *n*(%)	
Ascites	1 (0.8)
hepatic encephalopathy	1 (0.8)
gastrointestinal bleeding	0
Liver decompensation—OLTx, *n*(%)	0
Liver acute rejection in patients after liver transplantation, *n*(%)	0
HCC de novo during or after therapy, *n*(%)	1 (0.8)
Death, *n*(%)	1 (0.8)

RBV: Ribavirin; AE: adverse event; OLTx: Orthotopic liver transplantation; HCC: Hepatocellular carcinoma.

## Data Availability

Data supporting reported results can be provided upon request from the corresponding author.

## Supplementary Materials

The following supporting information can be downloaded at: www.mdpi.com/article/10.3390/v15030677/s1, Table S1. Reference values of laboratory parameters.
