# Supplementary material for: Rescue Therapy after Failure of HCV Antiviral Treatment with Interferon-Free Regimens
Source: Viruses. 2023 Mar 4;15(3):677. doi: 10.3390/v15030677 (PMC10055110; doi:10.3390/v15030677)
Supplement: Supplementary file 1 [file viruses-15-00677-s001.zip › viruses-2230471-supplementary.pdf]

Table S1 Suppl. Reference values of laboratory parameters.

Laboratory parameters	Reference values	Unit
ALT (Alanine transaminase)	<40	IU/l
Bilirubin	0,3–1,2	mg/dl
Albumin	3,5–5	g/dl
Creatinine	0,6–1,3	mg/dl
Hemoglobin	13–18	g/dl
Platelets	100–400	1000/ μl
